# In-silico interaction-resolution pathway activity quantification and application to identifying cancer subtypes

**DOI:** 10.1186/s12911-016-0295-2

**Published:** 2016-07-18

**Authors:** Sungwon Jung

**Affiliations:** Department of Genome Medicine and Science, Gachon University School of Medicine, Incheon, 21565 Republic of Korea

## Abstract

**Background:**

Identifying subtypes of complex diseases such as cancer is the very first step toward developing highly customized therapeutics on such diseases, as their origins significantly vary even with similar physiological characteristics. There have been many studies to recognize subtypes of various cancer based on genomic signatures, and most of them rely on approaches based on the signatures or features developed from individual genes. However, the idea of network-driven activities of biological functions has gained a lot of interests, as more evidence is found that biological systems can show highly diverse activity patterns because genes can interact differentially across specific molecular contexts.

**Methods:**

In this study, we proposed an in-silico method to quantify pathway activities with a resolution of genetic interactions for individual samples, and developed a method to compute the discrepancy between samples based on the quantified pathway activities.

**Results:**

By using the proposed discrepancy measure between sample pathway activities in clustering melanoma gene expression data, we identified two potential subtypes of melanoma with distinguished pathway activities, where the two groups of patients showed significantly different survival patterns. We also investigated selected pathways with distinguished activity patterns between the two groups, and the result suggests hypotheses on the mechanisms driving the two potential subtypes.

**Conclusions:**

By using the proposed approach of modeling pathway activities with a resolution of genetic interactions, potential novel subtypes of disease were proposed with accompanying hypotheses on subtype-specific genetic interaction information.

## Background

Since the emergence of high throughput genomic profiling techniques, genomic profile data became a primary source of information in recognizing the various statuses of complex diseases. Cancer is one of such complex diseases, where even tumors from the same tissue locations can have strikingly diverse molecular mechanisms for their origins. Such high heterogeneity in cancer is one of the main obstacles in treatment, as different driving mechanisms may require different therapeutic approaches to repair their abnormality. For this reason, identifying subtypes of cancer with different functional mechanisms is very important for improving their successful diagnosis and treatment.

One of the popular approaches to recognizing the subtypes of cancer is clustering the gene expression data of patient samples (for example, [1–7]), as expression data can give a comprehensive snapshot of transcription activities for whole genes. Many clustering studies consider each gene as a feature for clustering, assuming the expression levels of individual genes are factors that discriminate the different subtypes of cancer. However, the main drawback of such approaches is that they focus on individual genes, while a set of interacting genes constitutes a functional module in many real biological systems. For this reason, using individual genes as features often suffer with the issue of low reproducibility, which indicates the expression levels of genes reflect only some part of discrepancy residing between different subtypes.

In order to overcome such limitation, utilizing known pathway information together with the expression data can be a promising approach. Considering that a joint probability distribution of a set of variables can give a comprehensive picture of its pattern, an ideal approach is modeling the joint probability distribution that describes the combinatorial gene expression levels within a pathway. However, this approach is not practical due to the complexity of the model to represent the joint probability distribution, and the lack of available data to infer such complex models with sufficient reliability. Hence, most of the methods to utilize pathway information focus on specific features of pathways rather than considering the complete joint probability distributions. Characterizing individual samples with pathway information and applying it to clustering achieved limited success, while there is a recent study that proposed a method called PARADIGM [8], which infers patient-specific gene activities from multi-dimensional genomic data using known genetic interactions from pathways. PARADIGM can convert multiple genomic data of a gene from a sample into a single aggregated value called IPA, which represents the summarized activity level of the gene for the sample and it is evaluated in consideration of genetic interactions from pathway information. The computed IPA values of genes can be used for clustering instead of their raw expression values, but it still represents the activity levels of individual genes rather than the activity levels of pathways.

In this study, a method was proposed to compute the dissimilarity between two gene expression samples based on features that represent pathway activity patterns. Unlike conventional methods, our proposed method converts a gene-level matrix (for example, gene expression matrix) to a pathway-level matrix, where each cell in the matrix represents a pathway activity pattern for a sample. We applied the proposed sample dissimilarity measure to clustering of cancer samples, where the RNA-Seq data of 267 melanoma patients from The Cancer Genome Atlas (TCGA) was clustered based on their pathway activities. Two patient groups of potential subtypes were identified with clear difference in their survival patterns, where they were associated with different stages of melanoma. Investigation on selected pathway activity patterns across two patient groups suggested hypotheses on different functional mechanisms driving two potential subtypes.

## Methods

Our approach is based on an assumption that the activity pattern of a pathway for a sample can be represented with the probability distribution of the genetic network likelihoods from the pathway, which is computed from the given gene expression data. *A sample pathway activity vector (PAV)*, which represents the comprehensive picture of all pathway activities of a single sample, is represented as a collection of pathway activities for all pathways for the sample. *A pathway activity vector distance (PAVd)* is proposed as a discrepancy measure between two sample pathway activity vectors, which represents the dissimilarity between the two samples from the perspective of pathways. As *PAVd* is a distance metric (this will be discussed in the following subsections), arbitrary clustering methods and cluster validation indexes can be used for clustering and quality evaluation. Details of this formulation will be given in the following subsections.

### Pathway activity distribution

We compute the activity of a pathway for a sample by approximating the probability distribution of genetic networks from the pathway. Specifically, the *pathway activity distribution* Pr(*PA*_*i*_, *s*_*j*_) of a pathway *PA*_*i*_ for a sample *s*_*j*_ is computed from the following steps:

**Step 1)** Consider *PA*_*i*_ as a discrete random variable that has a finite set of *N* genetic network structures *g*_*1*_, *g*_*2*_, …, *g*_*N*_ as its possible values.

**Step 2)** Compute the likelihood *L*_*k*_ = *P*(*g*_*k*_|*s*_*j*_) for each genetic network *g*_*k*_ for sample *s*_*j*_. The collection of likelihoods [*L*_*1*_, *L*_*2*_ … *L*_*N-1*_, *L*_*N*_] for *N* genetic network structures constitutes the pathway activity distribution Pr(*PA*_*i*_, *s*_*j*_) of pathway *PA*_*i*_ for a sample *s*_*j*_.

Compared to the idea of computing a single scalar-valued activity for a pathway, our approach of computing the pathway activity as a probability distribution is a generalized version of such idea. From this generalization of considering multiple genetic networks, it is expected to achieve more reliable measurement of pathway activities than the idea of computing a single scalar-valued activity.

**Fig. 1:**
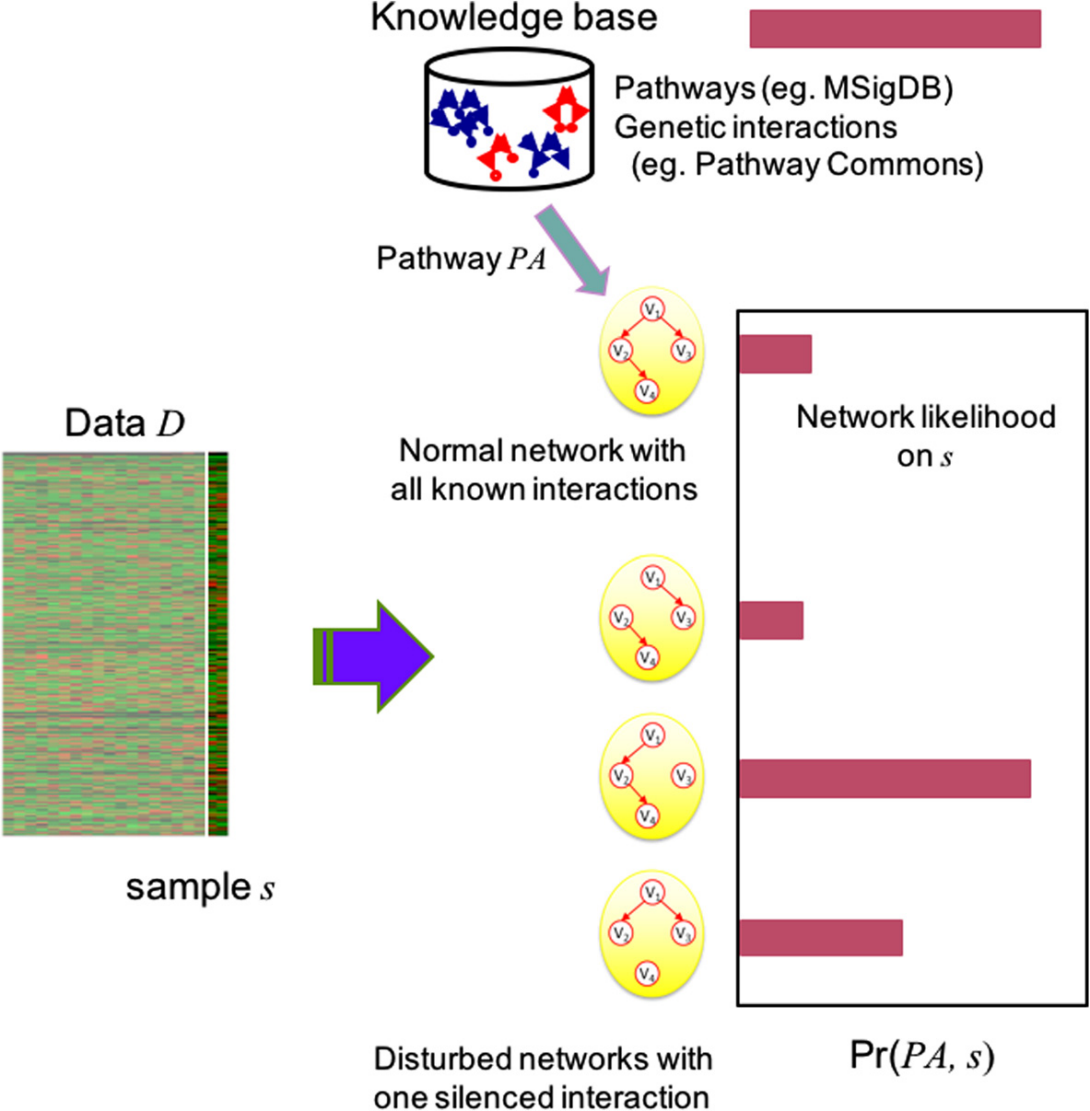
A schematic diagram of computing pathway activity distribution

In computing the pathway activity distribution Pr(*PA*_*i*_, *s*_*j*_), we utilize existing knowledge base on pathways and gene regulatory interactions. Instead of enumerating all possible genetic network structures for *PA*_*i*_, a selected list of candidate genetic networks are considered in reference with a knowledge base of choice, to compute Pr(*PA*_*i*_, *s*_*j*_). The list of candidate networks will include one genetic network of normal status, where we assume that a normally acting pathway has all known genetic interactions functioning. From this normal network with all known genetic interactions, we assume that eliminating an interaction can represent a disturbed status of the pathway. This assumption is based on an idea that disruption of a pathway by external variables (for example, regulation by miRNA, epigenetic changes, gene copy number variation) can be represented with *silencing* certain genetic interactions within the pathway. As a result, a normal network and disturbed networks with one silenced interaction are considered as possible values of *PA*_*i*_. The schematic outline of this approach is illustrated in Fig. 1.

To compute the likelihood *L*_*k*_ = *P*(*g*_*k*_|*s*_*j*_) for each genetic network *g*_*k*_ for sample *s*_*j*_, we model a genetic network with a Bayesian network structure assuming discrete random variables as its nodes. The computation of likelihood is done using the Bayesian Dirichlet equivalence uniform (BDeu) scoring method [9]. However, the direct computation of likelihoods with only single sample yields uniform likelihoods for all samples as the BDeu scoring method considers each instance (sample) of variables with the same preference, especially with the uniform prior assumption. For this reason, we take an indirect approach to compute likelihoods, with the following formulation:

1 $$ P\left({g}_k\Big|{s}_j\right)=\frac{BDeu\left({g}_k\Big|D\right)}{BDeu\left({g}_k\Big|D-\left\{{s}_j\right\}\right)} $$

where *D* represents the collection of all samples. Even though we use the Bayesian network model assuming discrete random variables, our formulation is independent of model choices. Thus other network and random variable models can be also used as long as the likelihood of a network structure can be computed based on the model of preference.

### Sample pathway activity vector

The sample pathway activity vector *PAV*(*s*_*j*_, ***PA***) of sample *s*_*j*_ for a set of *A* pathways ***PA*** is defined with a vector of pathway activity distributions as follows:

2 $$ PAV\left({s}_i,PA\right)=< \Pr \left(P{A}_1,{s}_i\right), \Pr \left(P{A}_2,{s}_i\right),\dots, \Pr \left(P{A}_A,{s}_i\right)> $$

For *A* pathways and *S* samples, the pathway activity distribution matrix *R* is defined as a *A* × *S* matrix, where a cell *R*(*i*, *j*) corresponds to a pathway activity distribution Pr(*PA*_*i*_, *s*_*j*_) of a pathway *PA*_*i*_ for a sample *s*_*j*_. In other words, *R* is a collection of column vectors *PAV*(*s*_*j*_, ***PA***) for *S* samples.

### Discrepancy measure between two sample pathway activity vectors

If a column vector in a pathway activity distribution matrix *R* is a scalar-valued vector with each pathway activity represented with a scalar value, conventional distance measures (such as Euclidean distance) assuming ordinary scalar-valued vectors can be used to evaluate the discrepancy between two samples. In our approach of representing the pathway activity with a discrete probability distribution Pr(*PA*_*i*_, *s*_*j*_), the representation of sample pathway activity *PAV*(*s*_*j*_, ***PA***) of a sample *s*_*j*_ for a set of *A* pathways is a vector of probability distributions as shown in Eq. (2). As each element of a pathway activity vector *PAV*(*s*_*j*_, ***PA***) is a probability distribution rather than a scalar value, a new method is necessary to compute the distance between two vectors of probability distributions *PAV*(*s*_*l*_, ***PA***) and *PAV*(*s*_*m*_, ***PA***) from two samples *s*_*l*_ and *s*_*m*_.

We designed a new distance measure *pathway activity vector distance* (*PAVd*) to compute the distance between two vectors of discrete probability distributions *PAV*(*s*_*l*_, ***PA***) and *PAV*(*s*_*m*_, ***PA***), which is defined as follows:

3 $$ PAVd\left(\left(PAV\left({s}_l,PA\right)\right)\left|\right|PAV\left({s}_m,PA\right)\right)={\displaystyle {\sum}_{i=1}^A\sqrt{JS\left( \Pr \left(P{A}_i\Big|{s}_l\right)\right)\left|\right| \Pr \left(P{A}_i\Big|{s}_m\right)}} $$

where *JS* is the Jensen-Shannon divergence. The Jensen-Shannon divergence is a symmetrized version of the Kullback-Leibler divergence, and a popular method of measuring the similarity between two probability distributions. Note that *PAVd* is a metric, as it satisfies the four required properties – non-negativity, identity of indiscernibles, symmetry and triangle inequality.

**Corollary 1.***PAVd* satisfies a property, the non-negativity.

*Proof.* The Jensen-Shannon divergence *JS* of two probability distributions is a non-negative value.

4 $$ \therefore PAVd\left(PAV\left({s}_l,PA\right)\left|\right|PAV\left({s}_m,PA\right)\right)={\displaystyle {\sum}_{i=1}^A\sqrt{JS\left( \Pr \left(P{A}_i\Big|{s}_l\right)\left|\right| \Pr \left(P{A}_i\Big|{s}_m\right)\right)}}\ge 0 $$

**Corollary 2.***PAVd* satisfies a property, the identify of indiscernibles.

*Proof. PAVd*(*PAV*(*s*_*l*_, *PA*) || *PAV*(*s*_*m*_, *PA*)) is a sum of non-negative values from Eq. (3). Thus, *PAVd*(*PAV*(*s*_*l*_, *PA*) || *PAV*(*s*_*m*_, *PA*)) = 0 requires *JS*(Pr(*PA*_*i*_ | *s*_*l*_) || Pr(*PA*_*i*_ | *s*_*m*_)) to be 0 for all *i*. As the square root of the Jensen-Shannon divergence is a metric [10, 11], *JS*(Pr(*PA*_*i*_ | *s*_*l*_) || Pr(*PA*_*i*_ | *s*_*m*_)) = 0 if and only if Pr(*PA*_*i*_ | *s*_*l*_) = Pr(*PA*_*i*_ | *s*_*m*_). If Pr(*PA*_*i*_ | *s*_*l*_) = Pr(*PA*_*i*_ | *s*_*m*_) for all *i*, then *PAV*(*s*_*l*_, *PA*) = *PAV*(*s*_*m*_, *PA*).

5 $$ \therefore PAVd\left(PAV\left({s}_l,PA\right)\left|\right|PAV\left({s}_m,PA\right)\right)=0\;\mathrm{if}\;\mathrm{and}\;\mathrm{only}\;\mathrm{if}\;PAV\left({s}_l,PA\right)=PAV\left({s}_m,PA\right). $$

**Corollary 3.***PAVd* satisfies a property, symmetry.

*Proof.* The Jensen-Shannon divergence *JS* is a symmetrized version of the Kullback-Leibler divergence.

6 $$ \therefore PAVd\left(PAV\left({s}_l,PA\right)\left|\right|PAV\left({s}_m,PA\right)\right)= PAVd\left(PAV\left({s}_m,PA\right)\left|\right|PAV\left({s}_l,PA\right)\right) $$

**Corollary 4.***PAVd* satisfies a property, triangle inequality.

*Proof.* Consider three sample pathway activity vector *PAV*(*s*_*l*_, *PA*), *PAV*(*s*_*m*_, *PA*) and *PAV*(*s*_*n*_, *PA*). As the square root of the Jensen-Shannon divergence *JS* is a metric, the following is true for all *i*:

7 $$ \begin{array}{l}\kern14.36em \sqrt{JS\left( \Pr \left(P{A}_i\Big|{s}_l\right)\left|\right| \Pr \left(P{A}_i\Big|{s}_n\right)\right)}\\ {}\le \sqrt{JS\left( \Pr \left(P{A}_i\Big|{S}_l\right)\left|\right| \Pr \left(P{A}_l\Big|{s}_m\right)\right)}+\sqrt{JS\left( \Pr \left(P{A}_i\Big|{s}_m\right)\left|\right| \Pr \left(P{A}_i\Big|{s}_n\right)\right)}\end{array} $$

Thus, the following is also true:

8 $$ \begin{array}{l}{\displaystyle {\sum}_{i=1}^A\sqrt{JS\left( \Pr \left(P{A}_i\Big|{S}_l\right)\left|\right| \Pr \left(P{A}_i\Big|{s}_n\right)\right)}}\\ {}\le {\displaystyle {\sum}_{i=1}^A\sqrt{JS\left( \Pr \left(P{A}_i\Big|{S}_l\right)\left|\right| \Pr \left(P{A}_i\Big|{s}_m\right)\right)}}+{\displaystyle {\sum}_{i=1}^A\sqrt{JS\left( \Pr \left(P{A}_i\Big|{S}_m\right)\left|\right| \Pr \left(P{A}_i\Big|{s}_n\right)\right)}}\end{array} $$

9 $$ \begin{array}{l}\kern1.56em \therefore PAVd\left(PAV\left({s}_l,PA\right)\left|\right|PAV\left({s}_n,PA\right)\right)\\ {}\le PAVd\left(PAV\left({s}_l,PA\right)\left|\right|PAV\left({s}_m,PA\right)\right)+ PAVd\left(PAV\left({s}_m,PA\right)\left|\right|PAV\left({s}_n,PA\right)\right)\end{array} $$

**Theorem 1.***PAVd* is a distance metric.

*Proof.* From Corollary 1 to 4, *PAVd* satisfies the four properties of metric.

By using this distance metric *PAVd* with conventional clustering algorithms, we can group samples based on the sample pathway activities.

### Utilizing pathway information

We collected 1932 filtered gene sets of canonical pathways, Gene Ontology (GO) biological process and molecular functions from MSigDB [12], where each gene set has up to 50 genes, and used them as pathways in our study. The gene sets from MSigDB do not include genetic interaction information. For genetic interaction information, 854,464 human genetic interactions were obtained from Pathway Commons [13], and genes in each pathway were interconnected based on the obtained genetic interactions.

### Analysis of TCGA melanoma RNA-Seq data

We obtained the RNA-Seq data of 267 melanoma patients from TCGA. The normalized gene-level transcript counts were used for the analysis. The normalized counts of each gene were discretized into two values of 0 (not expressed) and 1 (expressed) using SIBER [14]. A sample pathway activity vector has been computed for each of the 267 patient samples, and a pathway activity distribution matrix *R* was built as a result. Using *PAVd* as a distance measure between sample pathway activity vectors that correspond to the columns of *R*, hierarchical clustering with complete linkage was applied to *R* to find groups of patients.

## Results

### Identification of two patient groups from the clustering result

**Fig. 2:**
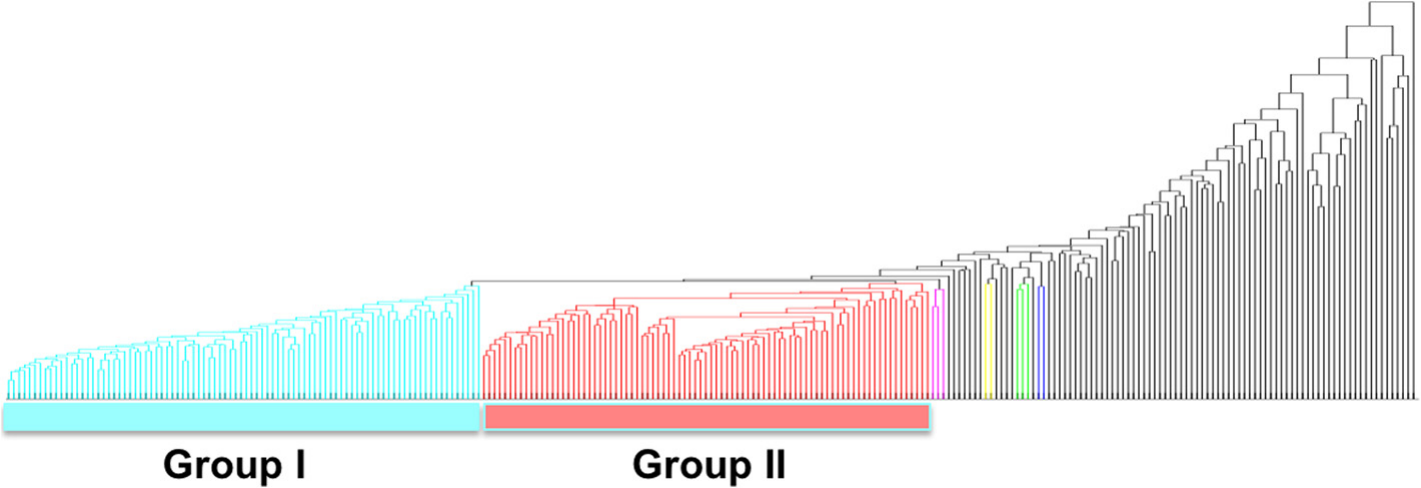
The result of applying hierarchical clustering to the 267 melanoma patient samples, using their computed sample pathway activities. Two groups of patients (Group I and II) were visually identified

Figure 2 shows the dendrogram from the result of applying hierarchical clustering to the pathway activity distribution matrix *R* of the TCGA melanoma RNA-seq data. After visual inspection, we identified two groups of patients, Group I and II. Based on the sample annotations regarding the stage of melanoma, we also computed what stages of melanoma cases are significantly enriched in each group of patients. Table 1 lists the number of patients in each group as well as the number of associated stage I-IV cases. From the entire 267 patients, 40 patients were classified as Stage I, and 18 of them were included in Group I (*p*-value = **0.0362**). In Stage I of melanoma, cancer has formed on skin, but tumor is not present in deep skin. Thus it represents relatively early stage of melanoma prognosis. Regarding Group II, there were 52 patients classified as Stage II among the 267 patients, and 24 of them were included in this group of patients (*p*-value = **0.0049**). When melanoma is in Stage II, it indicates that the tumor is in deeper skin than Stage I, with possible ulceration. Thus this stage indicates a more progressed status of melanoma. The absolute numbers of Stage I patients and II patients in these two groups may not be high, but Group I represents patients with earlier stage of (or less aggressive) melanoma while Group II represents patients with relatively later stage of (or more aggressive) melanoma. This observation can be confirmed in the following subsection.

**Table 1:** Identified two groups of patients and their melanoma stages

	Group I	Group II
Number of patients	90	85
Stage I	18 (*p* = **0.0362**)	7 (*p* = 0.9764)
Stage II	11 (*p* = 0.9779)	24 (*p* = **0.0049**)
Stage III	16 (*p* = 0.4976)	18 (*p* = 0.1623)
Stage IV	2 (*p* = 0.3258)	2 (*p* = 0.2889)

*p*-values are shown in the parentheses. (*p* < 0.05 are shown in boldface)

### Survival analysis of identified patient groups

**Fig. 3:**
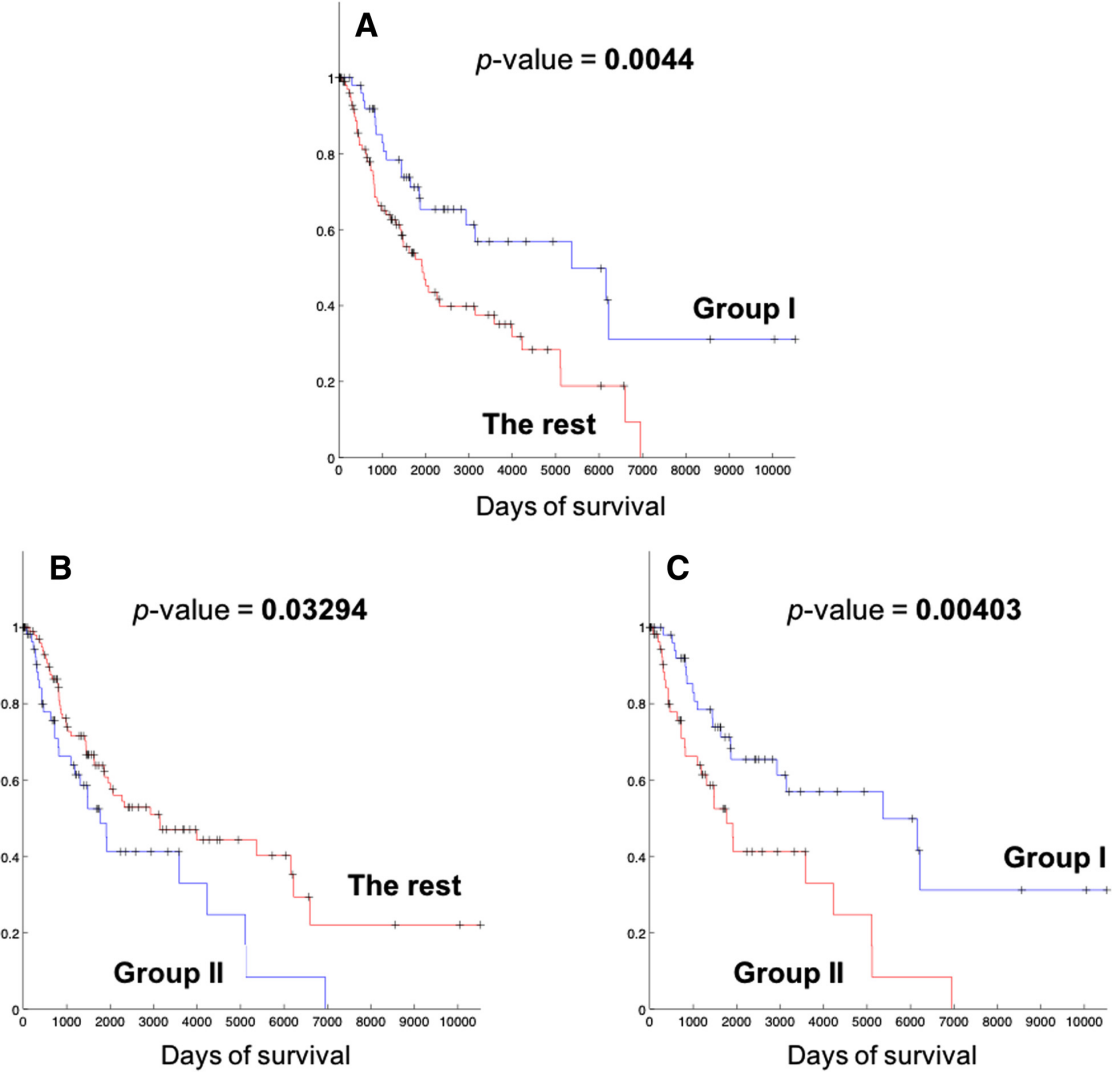
Kaplan-Meier (KM) plots of comparing survival patterns of Group I, II and the rest of the patients other than each group. Censored cases are indicated with marks. *P*-values were computed from the log-rank test of two survival patterns. **a** Comparison between Group I (90 patients) and the rest of the patients (177 patients). **b** Comparison between Group II (85 patients) and the rest of the patients (182 patients). **c** Comparison between Group I and II

**Fig. 4:**
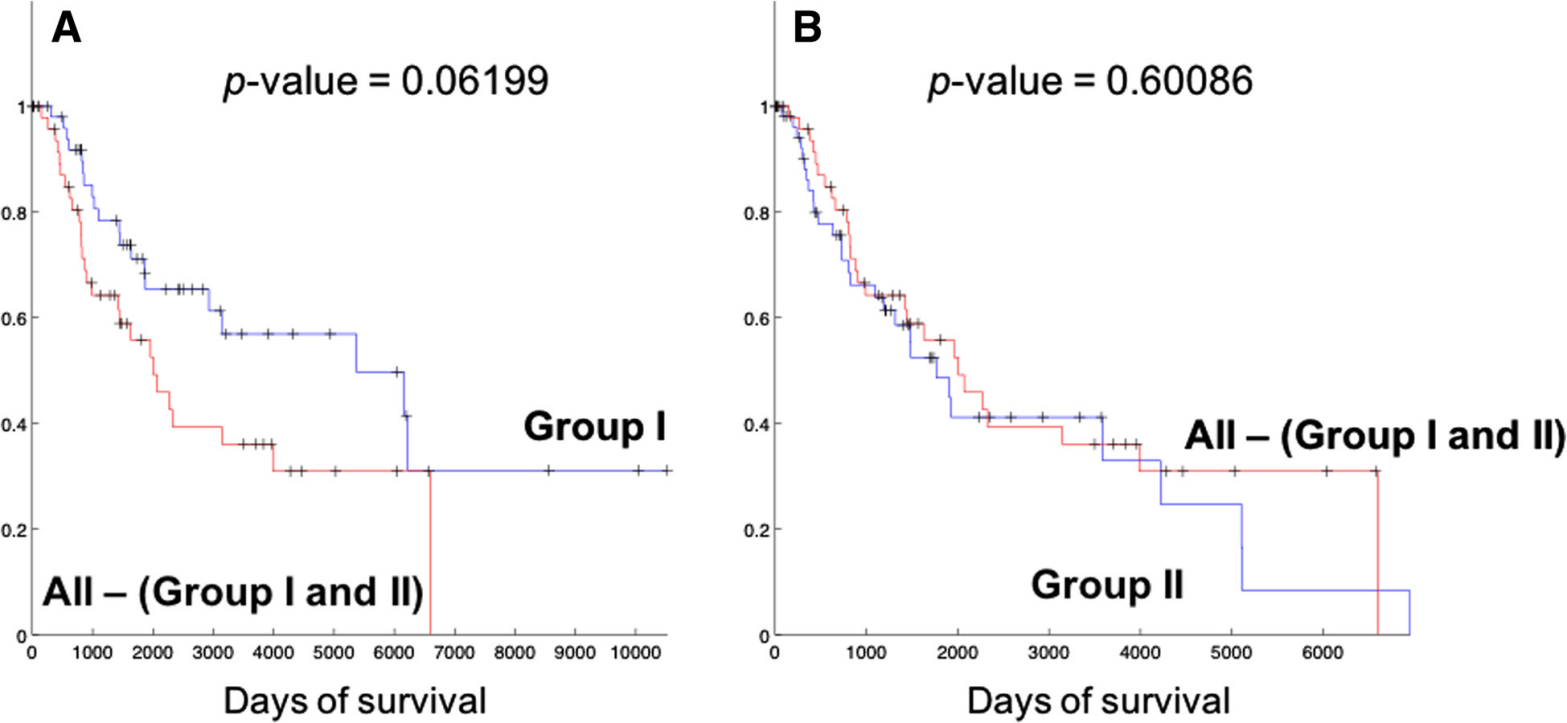
KM plots of comparing survival patterns of Group I and II with the other patients that do not belong to either Group I or II (All – (Group I and II)). Censored cases are indicated with marks. *P*-values were computed from the log-rank test of two survival patterns. **a** Comparison of Group I with patients that do not belong to either Group I or II. **b** Comparison of Group II with patients that do not belong to either Group I or II

We compared the survival lengths of patients in each group, and Kaplan-Meier (KM) curves from the comparison are shown in Fig. 3. Table 2 also lists the survival statistics on patient groups including Group I and II. From Fig. 3(a) and Table 2, it is clear that patients in Group I show better prognosis than the rest of the patients (statistical significance *p*-value = **0.0044**), with much longer median survival length (4254 days) than the rest of the patients. Patients in Group II have relatively shorter survival (median survival length of 1625.5 days) than the rest of the patients, but the main difference is with the patients in Group I (Fig. 3(c)) rather than with the patients other than Group I and II (All patients – (Group I and II patients), Fig. 4(b)). In comparison, the patients of Group I still show longer survival patterns than the patients that do not belong to the two groups (Fig. 4(a)). This suggests that Group I is a distinguished patient group compared to other patients, with clearly longer survival lengths, while Group II may have different biological mechanisms of melanoma compared to other patients while they do not show clearly different survival patterns.

**Table 2:** Survival statistics of identified patient groups

Patient group	Number of patients	Median survival (days)	Comparison versus the rest of the patients	
			Hazard ratio	*p*-value (log-rank test)
Group I	90	4,254	0.6298	**0.0044**
Group II	85	1,625.5	2.3281	**0.03294**
All – (Group I ∪ Group II)	92	1,982	0.7150	0.46775

*p* < 0.05 are shown in boldface

## Discussion

We compared the pathway activity patterns between Group I and II, and two pathways with distinguished activity patterns were selected for further investigation. The first pathway was a GO gene set *Regulation of Cell-Cell Adhesion* with one genetic interaction from Pathway Commons, and the second pathway was a *DNA Fragmentation* pathway with eight genetic interactions.

### Difference between Group I and II, based on regulation of cell-cell adhesion

**Fig. 5:**
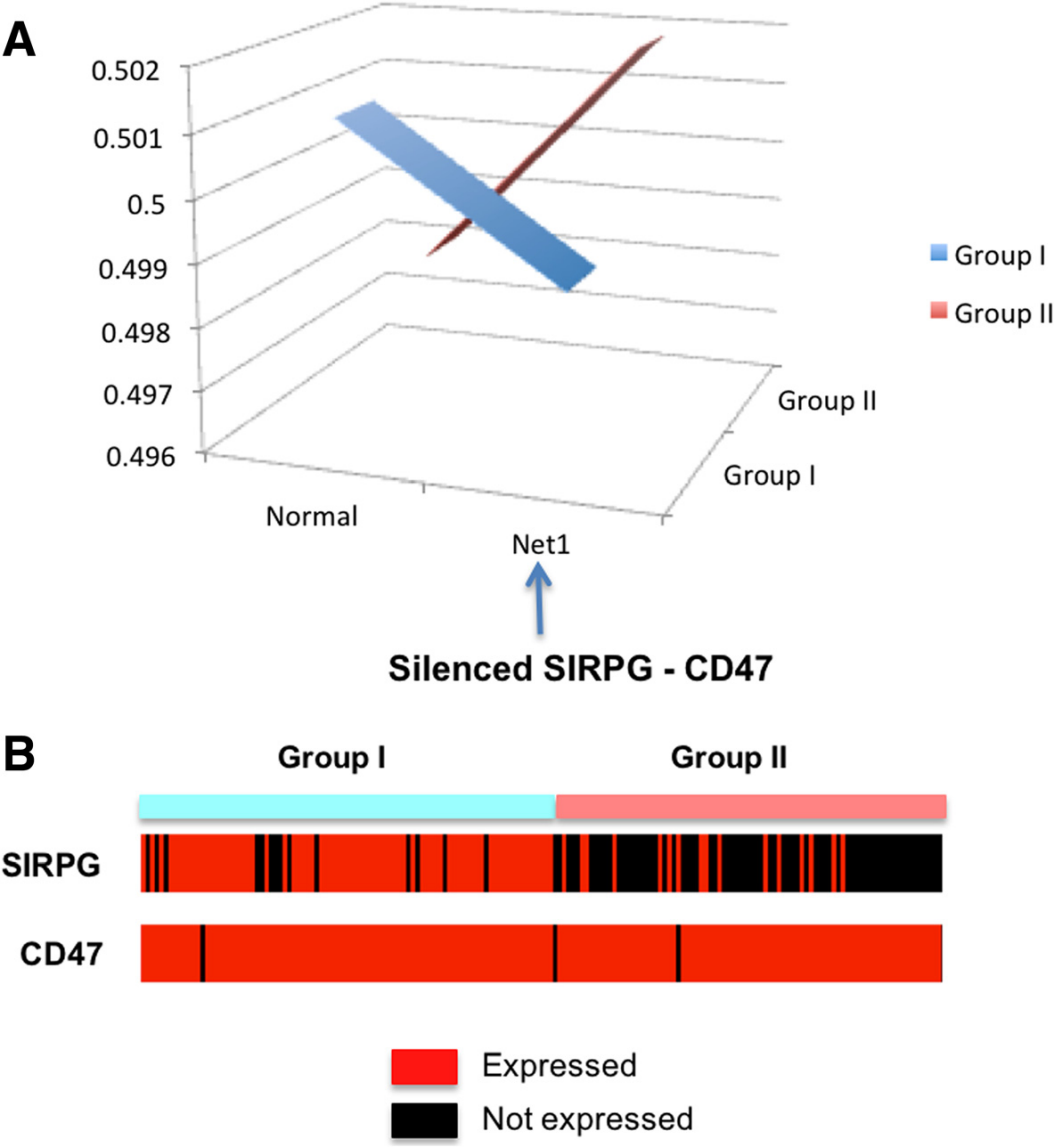
The *Regulation of Cell-Cell adhesion* pathway showing distinguished activity patterns between Group I and II. **a** Averaged pathway activity patterns for each Group I and II. “Normal” indicates the average likelihood of a genetic network with all interactions, across the samples of each group. “Net1” indicates the likelihood of a genetic network with a missing interaction SIRPG – CD47. **b** The discretized expression levels of SIRPG and CD47 across Group I and II

From each group, we computed the average pathway activity distribution Pr(*Regulation of Cell-Cell adhesion*, Group) by averaging the likelihoods of considered genetic networks across the samples within the group. Figure 5(a) shows the average activity pattern of the *Regulation of Cell-Cell adhesion* pathway, where Group I patients have higher likelihood of “Normal” genetic network while Group II patients have higher likelihood for a network with a missing SIRPG – CD47 interaction. The *Regulation of Cell-Cell adhesion* pathway is involved in cell-cell adhesion biology, which is a mechanism to bind a cell to a surface, such as an extracellular matrix or another cell. CD47 is a ligand for the SIRP protein family, and SIRP-gamma can bind to CD47. As mentioned earlier, the tumors from the Group II patients are more advanced (or aggressive) melanoma, where cancer cells show more break-in through skin tissues. As can be seen in Fig. 5(b), the ligand CD47 shows consistent expression across two groups of patients while SIRPG is not expressed in Group II patients. This suggests that the regulation of cell-cell adhesion might have been disturbed with silencing of the SIRPG – CD47 interaction through inhibited SIRPG, and it can be one of the mechanisms that cause tumor cells on the surface of skin to break-in toward deeper placements in the later stage melanoma.

### Difference between Group I and II, based on DNA Fragmentation

**Fig. 6:**
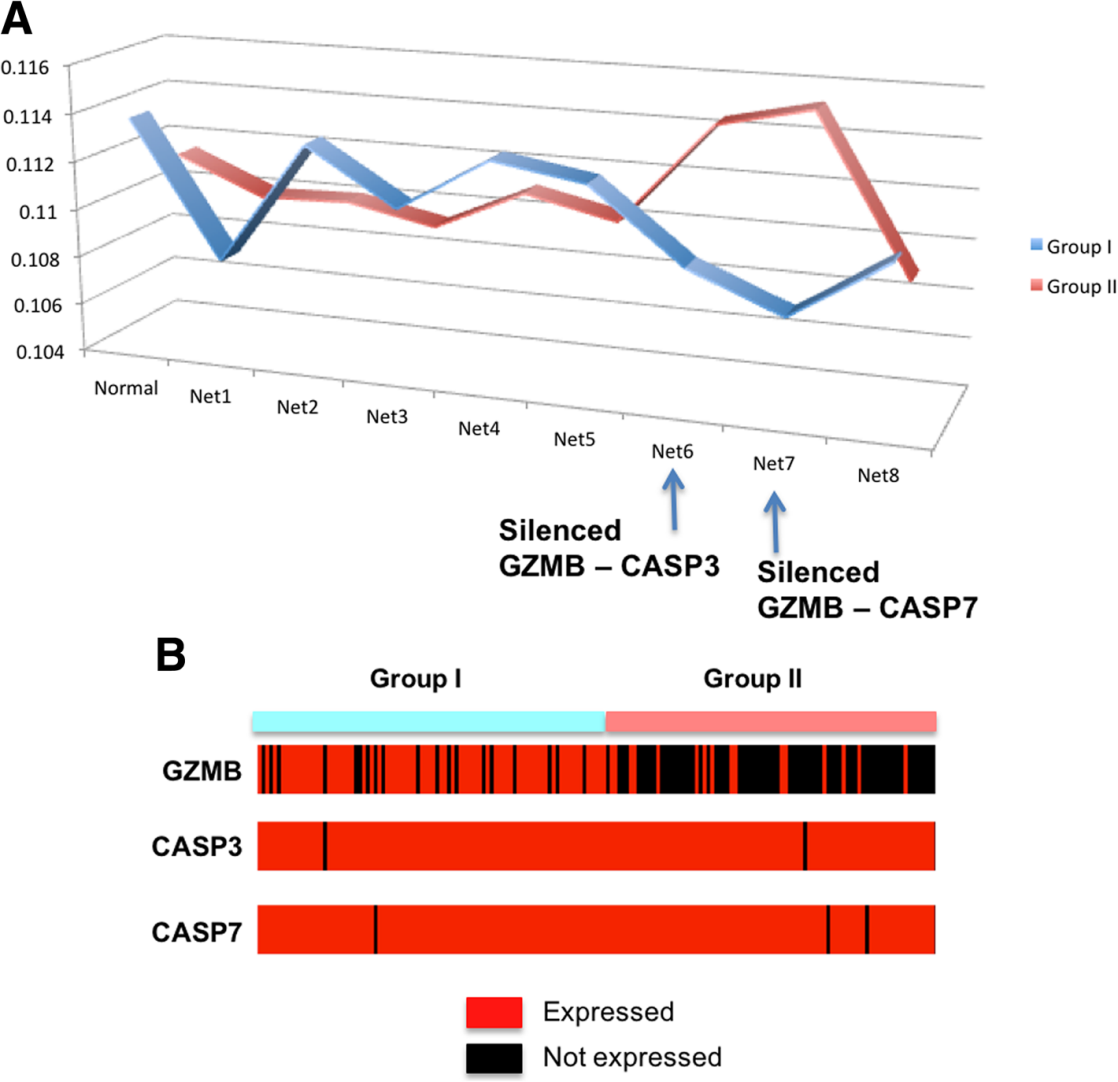
The *DNA Fragmentation* pathway showing distinguished activity patterns between Group I and II. **a** Averaged pathway activity patterns for each Group I and II. “Normal” indicates the average likelihood of a genetic network with all interactions, across the samples of each group. “Net(number)” indicates the likelihood of a genetic network with a missing interaction. “Net6” is a network with a silenced GZMB – CASP interaction, and “Net7” is a network with a silenced GZMB – CASP7 interaction. **b** The discretized expression levels of GZMB, CASP3 and CASP7 across Group I and II

We also compared the average pathway activity patterns of the *DNA Fragment* pathway between Group I and II. Figure 6(a) shows the average activity distribution of the *DNA Fragmentation* pathway, where Group I patients have higher likelihood of “Normal” genetic network. This suggests that the genetic interactions in the *DNA Fragmentation* pathway are better preserved in Group I patient tumors than the case of Group II, indicating Group II patients may have more abnormal activities of the DNA fragmentation mechanism. The DNA fragmentation pathway is one of the mechanisms that can be utilized during the immune response process, where immune cells send signals into target cells and cause apoptosis through the fragmentation of DNA in the target cells. From the pathway activity patterns in Fig. 6(a), two genetic network cases with two silenced genetic interactions (Net6 with a missing GZMB – CASP3 interaction, and Net7 with a missing GZMB – CASP7 interaction) are assigned with higher likelihoods from Group II than Group I. GZMB is expressed by cytotoxic T lymphocytes (CTL) and natural killer (NK) cells, and it is crucial for the rapid induction of target cell apoptosis. CASP3 and CASP7 are caspases, and their sequential activation plays a central role in the execution-phase of cell apoptosis. From Fig. 6(b), CASP3 and CASP7 are expressed from both of Group I and II patients, but GZMB is generally being inhibited in Group II patient tumors. These silenced interactions between GZMB and caspases genes suggest that the DNA fragmentation mechanism of immune cells has been restricted in Group II patients, resulting suppressed immune response. This hypothesis is consistent with the comparison of Group I and II, where Group II is enriched with later stage of melanoma cases and show worse survival patterns.

## Conclusions

We proposed a method to represent the complete pathway activity patterns of individual gene expression samples based on collected pathway information. By using each pathway activity as a feature rather than using individual genes, we formulated a distance measure *PAVd* that can compute the discrepancy between two gene expression samples in the scope of activity patterns of entire pathways. The proposed method of sample pathway activity quantification and computing distances based on the quantified activities has several potential benefits, as active biological mechanisms from individual samples can be more easily interpreted than using individual gene-based approaches. Compared to previous pathway evaluation methods such as GSEA [12] or PARADIGM, the proposed method of computing *PAV* provides unique functional benefit of analyzing single-sample pathway activities with a resolution of genetic interactions (Table 3), while *PAV* still needs to be extended to incorporate multiple types of genomic data. By applying the proposed method to cluster gene expression data of melanoma patients, we identified two potential subtypes of melanoma with distinguished pathway activity patterns. The two identified groups of patients showed distinctive survival patterns, and we suggested two hypotheses on biological mechanisms that can distinguish and potentially drive the two subtypes. This was possible because of our novel formulation on pathway activity considering genetic interactions, and we believe that there are much more potential applications of this approach as all components of the analysis – pathways, genetic interactions and etc. – are defined based on probabilistic models and can be easily extended with additional features. The two identified patient groups from this study correlate with stages of the disease, but each group still includes patients with multiple different stages. This implies that disease-driving biological mechanisms can reside across different stages of disease progression, and the proposed method contributes to identify such mechanisms.

**Table 3:** Functional difference of the proposed *PAV* compared to previous pathway evaluation methods

	Interaction-resolution	Multi-type data integration	Single sample evaluation
GSEA	No	No	No
PARADIGM	No	Yes	Yes
*PAV*	Yes	No	Yes

We are considering several directions for future studies. From our current study, we modeled the pathway activity patterns with a genetic network of normal status and genetic networks with only one missing interactions. Even though we showed a successful application of our formulation in this study, it will be definitely beneficial to consider genetic networks with more than one missing interactions, which leads to the generalization of the formulation with up to *K* missing interactions. We can also develop methods to quantitatively evaluate the abilities of pathways in discerning different clusters. As our formulation of the distance measure satisfies the properties of metric, we can incorporate the ideas of many conventional cluster validation indexes to evaluate the quality of clusters based on individual pathways. Lastly, we considered only gene expression data in our study, but integrating multiple types of genomic data in evaluation of pathway activity patterns and computing the effect of latent environment variables on pathways can be a promising direction to extend our current models.

### Ethics approval and consent to participate

Not applicable.

### Consent for publication

Not applicable.

### Availability of data and materials

All the data used in this article were obtained from public sources as cited.
